# Supplementary figures and images for: FOXM1 Modulation Alleviates Epithelial Remodeling and Inflammation in Eosinophilic Esophagitis
Source: bioRxiv. 2025 May 28:2025.05.25.655133. Preprint. [Version 1] doi: 10.1101/2025.05.25.655133 (PMC12154673; doi:10.1101/2025.05.25.655133)

# Supplemental figure S1

**A**

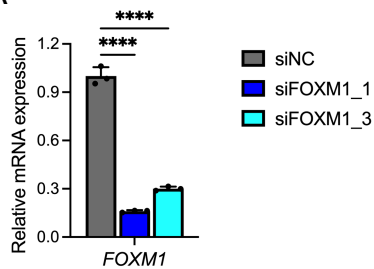

**B**

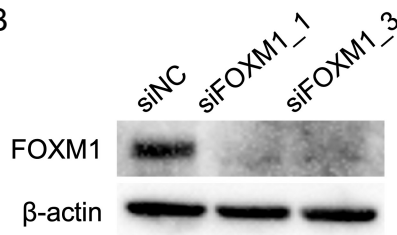

**C**

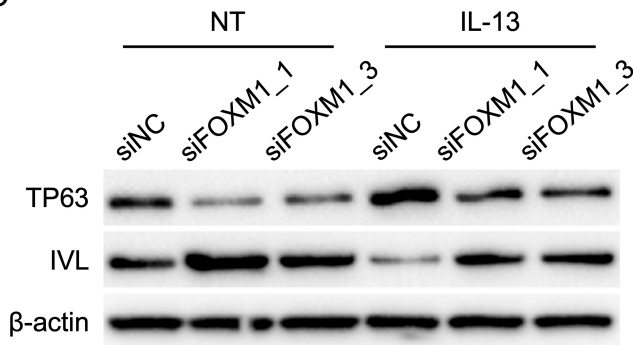

**D**

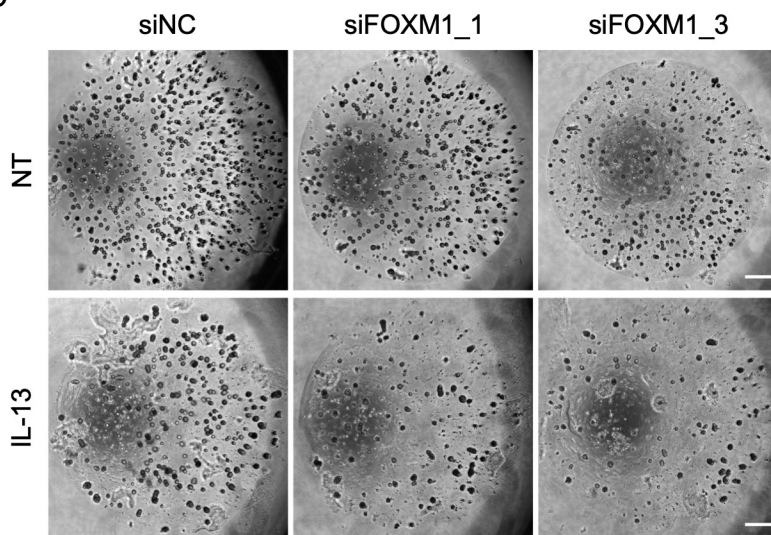

**E**

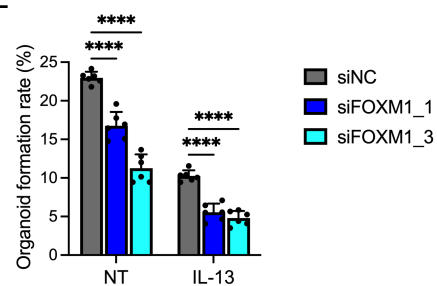

Supplement: Supplement 1 — (A-C) Quantitative RT-PCR for FOXM1 (n = 3) and representative images of immunoblot for FOXM1, TP63, and IVL in siFOXM1-transfected EPC2-hTERT cells in monolayer culture. Following the transfection, EPC2-hTERT cells were stimulated with or without IL-13 (10 ng/ml) in high-calcium (1.8 mM Ca2+) KSFM. Data are representative of three independent experiments and indicated as means ± SDs. (D and E) Representative phase contrast images of EPC2-hTERT organoids. After siRNA transfection, cells were reseeded and started the organoid culture. Organoids were stimulated with or without IL-13 (10 ng/ml) from day 7 to day 11 and organoid formation rate (OFR) was assessed at day 11. Scale bar, 1000 μm. OFR was defined as the number of organoids (≥50 μm) divided by the total seeded cells (n = 6). Data are representative of two independent experiments and indicated as means ± SDs. One-way analysis of variance (A and E) was utilized for statistics. ****P <0.0001. NC, negative control; NT, nontreated [file media-1.pdf]

# Supplemental figure S2

A

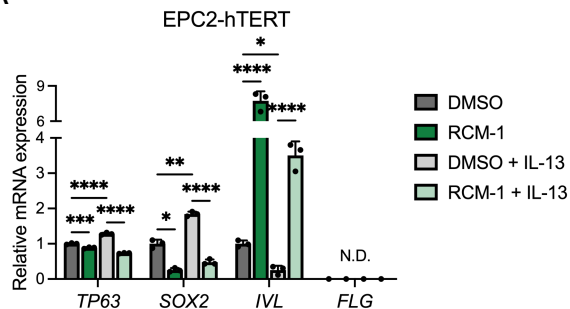

B

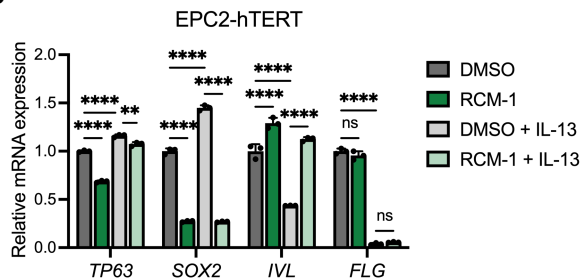

C

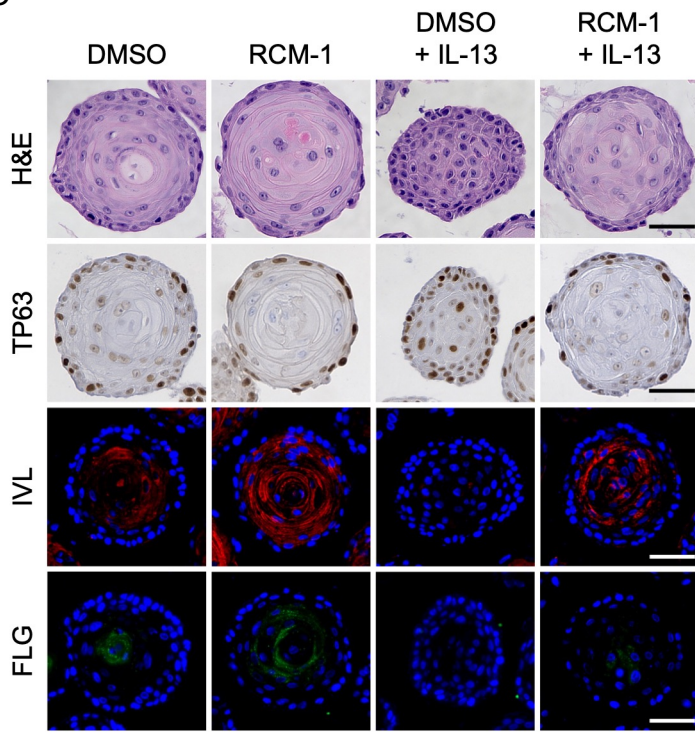

Supplement: Supplement 2 — (A) Quantitative RT-PCR in monolayer-cultured EPC2-hTERT cells. EPC2-hTERT cells were cultured in high-calcium (1.8 mM Ca2+) KSFM for 3 days along with or without 2 days of IL-13 (10 ng/ml) and RCM-1 (20 μM) treatment. (n = 3). (B and C) EPC2-hTERT cells were cultured for 7 days and then treated with or without IL-13 (10 ng/ml) and RCM-1 (10 μM) for 4 days. Day 11 organoids were subjected to quantitative RT-PCR (n = 3), hematoxylin and eosin (H&E) staining, immunohistochemistry for TP63, and immunofluorescence staining for IVL (red) and FLG (green) of the organoids. Representative images are shown. DAPI (blue). Scale bar, 50 μm. Data are representative of three independent experiments and indicated as means ± SDs. One-way analysis of variance (A and B) was utilized for statistics. *P <0.05, **P <0.01, ****P <0.001, ****P <0.0001. N.D., not detected, ns, not significant [file media-2.pdf]
